# Effects of Saccharomyces Cerevisiae Fermentation Products on the Microbial Community throughout the Gastrointestinal Tract of Calves

**DOI:** 10.3390/ani9010004

**Published:** 2018-12-21

**Authors:** Jianxin Xiao, Gibson M. Alugongo, Shoukun Ji, Zhaohai Wu, Shuangzhao Dong, Shengi Li, Ilkyu Yoon, Ruby Chung, Zhijun Cao

**Affiliations:** 1 State Key Laboratory of Animal Nutrition, Beijing Engineering Technology Research Center of Raw Milk Quality and Safety Control, College of Animal Science and Technology, China Agricultural University, Beijing 100193, China; dairyxiao@gmail.com (J.X.); maswayi@yahoo.com (G.M.A.); jishoukun@163.com (S.J.); wzh07128@163.com (Z.W.); zhaoddr@163.com (S.D.); lisheng0677@163.com (S.L.); 2 Diamond V Inc., Cedar Rapids, IA 52404, USA; iyoon@diamondv.com (I.Y.); rchung@diamondv.com (R.C.)

**Keywords:** calf, *Saccharomyces cerevisiae* fermentation product, bacterial community, species richness

## Abstract

**Simple Summary:**

*Saccharomyces cerevisiae* fermentation products (SCFP) are widely used for dairy cows and have been suggested to improve calf performance and health. However, the changes in microbial community along the gut in calves supplemented with SCFP have not been investigated extensively. This manuscript exhibited that calves supplemented with Saccharomyces cerevisiae fermentation products changed the microbial community of GIT and stimulated fibrolytic bacteria (*Lachnospiraceae and Ruminococcaceae*) colonization in early rumen and large intestine, respectively. Those alternations of microbiota in GIT might explain how SCFP works in calves.

**Abstract:**

The effect of *Saccharomyces cerevisiae* fermentation products (SCFP) on improving growth and health of calves could be attributed to the ability of SCFP to modulate the microbiota in the gastrointestinal tract (GIT). However, the changes in microbial community along the gut in calves supplemented with SCFP have not been investigated extensively. The aims of this study were to investigate the effect of SCFP on microbial communities in each sites of GIT using high-throughput sequencing technique. Fifteen Holstein male calves were used and randomly assigned to 1 of the 3 treatments including a calf starter containing 0 (Control, CON), 0.5 (SCFP1) or 1% SCFP (SCFP2, Original XPC, Diamond V, Cedar Rapids, IA, USA) of dry matter from day 4 to 56. The supplemented calves were fed with an additional 1 g/d SCFP (SmartCare, Diamond V, Cedar Rapids, IA, USA) in milk from day 2 to 30. Rumen fluid was sampled at day 28 of age via esophageal tube. All calves were slaughtered and gastrointestinal samples collected on day 56. Inclusion of SCFP increased the microbial species richness in the large intestine. The SCFP also affected the bacterial community at an early age in the rumen and later in rectum microbiota. Supplementation of SCFP stimulated colonization by fibrolytic bacteria (*Lachnospiraceae* and *Ruminococcaceae*) in rumen and large intestine, respectively. No differences were found between SCFP1 and SCFP2. This is the first study to analyze the effect of SCFP on bacterial community of the GIT microbiota in calves. The results provide the basic bacterial community information, which helps us understand the mechanism of action of SCFP for improving the health and performance of pre-weaning calf.

## 1. Introduction

The rumen bacteria are the most important microorganisms in the gastrointestinal tract (GIT), where they interact with the host, contributing to its performance and health [1]. Recent evidence shows that the colonization by microorganisms in rumen [2] and intestines [3,4] occurs immediately after birth, with some of the essential microbes involved in mature rumen function present as early as at day 1 [5]. Both liquid and solid feed are likely to influence the bacterial community in the fore [1] and hind gut [6,7] of pre-weaning calves. As an important functional part of diets, feed additives, are also effective in altering the gut bacterial community and related animal health and performance [8,9]. 

*Saccharomyces cerevisiae* fermentation products (SCFP) are common feed additives in the dairy industry. The yeast fermentation process produces biologically active compounds including oligosaccharides, organic acids, amino acids and peptides [10] which can improve the survival of calves under stress, resulting in greater profit margins [11]. Other benefits of SCFP in calves include improved ADG and body structure [12], enhanced VFA production [13] and a more anatomically and physiologically developed rumen [14]. Furthermore, SCFP have the potential to influence the microbial composition in the GIT. In mature animals, SCFP stimulated the growth of ruminal fiber-digesting bacteria (*Ruminococcus albus, Ruminococcus flavefaciens and Fibrobacter succinogenes*) and lactate-utilizing bacteria [15,16,17]. Saccharomyces cerevisiae fermentation products can be supplemented in milk, milk replacer, and/or calf starter. Supplementing SCFP in milk or milk replacer can ensure the consumption of the product from very early in life of the calf. We have previously demonstrated that SCFP can influence *Butyrivibrio* and *Prevotella* in the rumen fluid [18]. These calves were fed Original XPC (XPC; Diamond V, Cedar Rapids, IA, USA) and SmartCare (SC; Diamond V, Cedar Rapids, IA, USA) which are SCFPs supplemented in starter and milk (or milk replacer), respectively. However, the effect of supplementing SCFP in diets of calves on hindgut microbial community has not yet been fully elucidated. Therefore, to further understand whether and how SCFP modulate the microbial population along the GIT in pre-weaning Holstein calves, we investigated microbial composition at different taxonomic levels and in different sites in the first 56 days of life. 

## 2. Materials and Methods

### 2.1. Ethics Approval

The experimental design and procedures were executed according to the protocols approved by the Ethical Committee of the College of Animal Science and Technology, China Agricultural University (protocol number: 2013-5-LZ). Animal care and use strictly followed the Regulations for the Administration of Affairs Concerning Experimental Animals, National Committee of Science and Technology of China (14 November 1988) and Instructive Notions with Respect to Caring for Laboratory Animals, Ministry of Science and Technology of China (30 September 2006). 

### 2.2. Animal Trials

The experiment was conducted using 15 Holstein male calves, which were separated from their dams immediately after birth. All calves averaged 41.8 ± 2.5 kg BW and only calves with successful passive transfer of immunity were used (≥5.5 g/dL of serum total protein, determined by clinical refractometer 24 h after birth). The calves were raised in hutches with free access to calf starter and clean water from day 4. Straw was used as bedding and renewed weekly. All calves received 4 L of colostrum (Brix values ≥ 22%) within 1 h of birth, and thereafter pasteurized milk were fed twice daily at 800 and 1,500 h from day 2 to day 56, calves received 6 L/d of milk from day 2 to 10, 8 L/d from day 11 to 42, 6 L/d from day 43 to 49 and 4 L/d afterwards until weaning on day 56. Calves were randomly assigned to 1 of the 3 treatments including CON (no SCFP), SCFP1 (1 g/d SmartCare in the milk + 0.5% XPC in the starter), and SCFP2 (1 g/d SmartCare in the milk + 1% XPC in the starter). The XPC was incorporated in the texturized calf starter (19% CP; HONNEUR Nutritional Technology Co., Ltd., Beijing, China) from day 4 to day 56 while SmartCare was added into milk daily in the morning milk feeding from day 2 to day 30. The calves were weaned and harvested on day 56. SmartCare is a water-soluble product that can be supplemented to milk and XPC is a dry feed product that can be added in starter. The combination of these products is the basis for Diamond V’s dairy calf program during pre-weaning phase.

Ingredients and chemical composition of calf starter was identical among treatments with the exception of SCFP content, which was replaced as a part of corn germ meal. Nutrient composition of starter met or exceeded the requirements for pre-weaned Holstein calves [19], and was composed of steam flaked corn (33.1%), wheat bran (7.6%), canola meal (8.0%), extruded soybean (4.2%), soybean meal (14.3%), corn germ meal (8.5, 8.0 and 7.5% for CON, SCFP1 and SCFP2, respectively), rice powder (4.2%), milk powder (0.4%), DDGS (16.0%), CaCO_3_ (1.4%), CaHPO_4_ (1.0%), NaCl (0.7%), mycotoxin binder (0.1%), premix compound (0.5%) and XPC (0, 0.5 and 1% for CON, SCFP1 and SCFP2, respectively). Starter and water were offered *ad libitum* intake 1 h after milk feeding. 

### 2.3. Sample Collections

#### 2.3.1. Rumen Fluid Collection on Day 28 

Rumen fluid was collected on day 28 of age by a flexible esophageal tube (6 mm of inner diameter and 2 mm of wall thickness; Anscitech Co., Ltd., Wuhan, Hubei, China) from all 15 calves (five per treatment) 4 h after the morning milk feeding. The first 10 mL of rumen fluid was discarded to avoid saliva contamination. Rumen liquid sample (RL28) was obtained by filtering rumen fluid through four layers of cheesecloth, and 10 mL of the liquid (RL28) was immediately frozen in liquid nitrogen and stored at −80 °C until DNA extraction.

#### 2.3.2. Collection of Rumen and Intestine Contents on Day 56 

All calves were slaughtered and samples of gastrointestinal contents were collected on day 56 of age. After slaughtering, the abdominal cavity was immediately opened and each region of digestive tract (rumen, duodenum, cecum, and rectum) was isolated and tied off. The rumen solid (RS56) and liquid (RL56) fractions were obtained by squeezing the rumen digest samples through four layers of sterile cheesecloth. Rumen solid (RS56), RL28, RL56, intestinal contents from duodenum (DC56), cecum (CC56) and rectum (RC56) were separately placed into sterile tubes, snap-frozen in liquid nitrogen, and then stored at –80 °C pending further analysis. 

### 2.4. DNA Isolation and Illumina Hiseq Sequencing

Rumen and intestinal samples were sent to Beijing Computing Center (Beijing, China) for DNA extraction, 16S rRNA gene amplification and sequencing. DNA was extracted from 2.5 g rumen solid fraction, 400 µL rumen fluid fraction and duodenum contents, 0.5 g cecum contents and 200 mg rectum contents using QIAamp DNA Mini Kit (Qiagen, Hilden, Germany) following the manufacturer’s protocol. 

For illumina Hiseq sequencing, the V3 region of the 16S rRNA genes was amplified using primers 343F (5^′^-GATCCTACGGGAGGCAGCA-3^′^) and 534R (5^′^-GCTTACCGCGGCTGCTGGC-3^′^) with barcodes. The NEB Next Ultra DNA Sample Prep Kit (NEB, Ipswich, MA, USA) was used to generate sequencing libraries as per manufacturer’s instructions and standard Illumina sample-preparation protocol [20]. The Agilent Bioanalyzer 2100 system (Agilent Technologies, Palo Alto, CA, USA) and Qubit 2.0 Fluorometer (Life technologies, Grand Island, NY, USA) were used to evaluate the library quality and subsequently sequenced on an Illumina Hiseq 2500 platform. Paired-end reads with 250–300 bp were generated.

### 2.5. Data Processing and Analysis

#### 2.5.1. Quality Control and Paired-End Reads Assemblies

Quality sequences with a score of >30 were obtained by FastQC (Version 0.11.3, Babraham Bioinformatics, Babraham, UK). In order to obtain intact amplicons, paired-end reads with no mismatches from the original DNA fragments were merged using FLASH (Version 1.2.7, Adobe, San Jose, CA, USA) [21]. Concatenated and chimeric sequences were detected and subsequently filtered out by USEARCH (Version 6.1, Robert Edgar, Tiburon, CA, USA). Trimmed sequences were uploaded to QIIME (Version 1.8.0, Gregory Caporaso, Boulder, CO, USA) for further analysis.

#### 2.5.2. OTU Cluster and Species Annotation

Sequence analysis was conducted using QIIME pipeline (Version 1.8.0, Gregory Caporaso, Boulder, CO, USA) [22]. Trimmed sequences were assigned to different samples based on barcodes and binned into operational taxonomic units (OTUs) by clustering sequences with a 97% similarity using the UCLUST software (Version 1.2.22, Robert Edgar, Tiburon, CA, USA) after removal of barcode and primers. The Greengene database and RDP classifier were used to classify the generated OTUs [23,24]. To reduce systematic variation and ensure the compatibility of the species diversity between the samples [25], the threshold of standardized sequences was set at 70,000 sequences (corresponding to the number of sequences in the minimum data set).

#### 2.5.3. Data Analysis

Alpha diversity indices (ACE, Chao1, Shannon and Simpson) were determined using QIIME pipeline (Version 1.8.0) [22]. The beta diversity indices, principal coordinate analysis (PCoA) and ANOSIM analysis between samples were determined based on Bray-Curtis metrics with Vegan package in R (Version 2.4-1, Microsoft, Redmond, WA, USA) [26]. Heatmaps were produced by heatmap packages exhibiting differences in major bacterial community OTUs (top 80 OTUs) among dietary groups at different sampling sites (RL28, RL56, RS56, DC56, CC56 and RC56). Venn diagrams were created to characterize the overlap of OTUs present among dietary groups within each sampling site using the *gplots* package in R [27]. Singleton OTUs were removed from this analysis. Microbial composition graphs and bacterial abundance were subsequently generated under different classification levels. One rumen liquid sample, from CON and SCFP2 each collected at day 28 (RL28) was excluded from all the above analysis because they contained an unusually low number of sequences and huge differences within group. 

Treatment effects were assessed using all 15 calves (88 samples). Variables of alpha diversity indices and bacterial abundance were analyzed separately by each sampling site using GLM procedure of SAS 9.2 (SAS Inst. Inc., Cary, NC, USA) with the fixed effect of treatment and the random effect of calf nested within treatment. Significant differences were indicated at *p* value ≤ 0.05. Comparison between treatments was carried out in SAS 9.2 (SAS Inst. Inc., Cary, NC, USA) using contrast statement to test (1) CON vs. SCFP (SCFP1 and SCFP2), and (2) SCFP1 vs. SCFP2. The Bonferroni correction was used when comparing the taxa abundance between treatments.

### 2.6. Nucleotide Sequence Accession Numbers

The identified sequences were deposited in the database at NCBI under SRX1744697, SRX1744731, SRX1744733, SRX1744734, SRX1744735 and SRX1744736.

## 3. Results

### 3.1. Sequences and OTUs

For each individual (15 male calves), the samples included RL28, RL56, RS56, DC56, CC56 and RC56. After sequencing and sequence trimming, a total number of 14,556,361 quality reads were obtained from three treatments (CON, 4,926,750; SCFP1, 4,802,409; SCFP2, 4,827,202). The average reads per sample were 161,737. Among the 88 samples, an average of 1582, 1563 and 1588 OTUs were detected for CON, SCFP1 and SCFP2, respectively. The average and range of sequences and OTUs of each treatment in different sites were presented in Table 1. Good’s coverage was high with an average of 0.99 across all samples, which imply sufficient depth in sequencing in the present study.

### 3.2. Impact of SCFP on Microbiota

#### 3.2.1. Microbial Richness and Diversity

**Figure 1:**
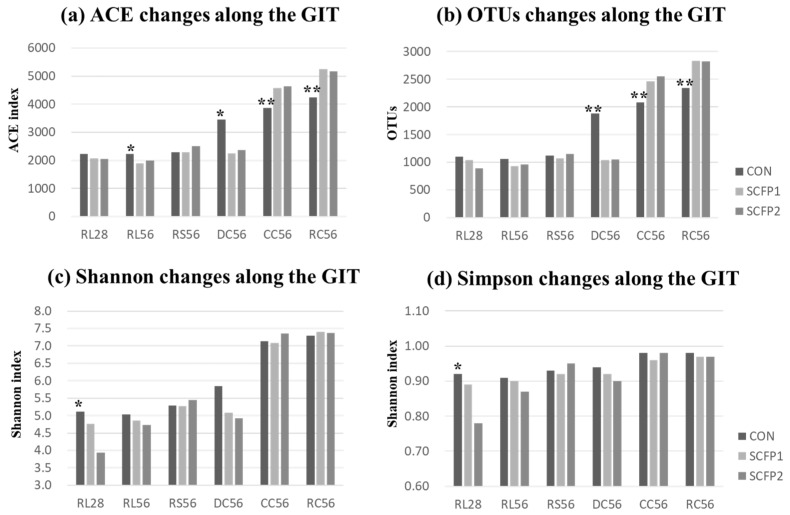
The difference of average (**a**) ACE, (**b**) OTU numbers, (**c**) Shannon and (**d**) Simpson indices values of CON, SCFP1 and SCFP2 along the GIT. CON = No SmartCare and XPC; SCFP1 = 1 g/head/d SmartCare in milk + 0.5% XPC in the starter grains; SCFP2 = 1 g/head/d SmartCare in milk + 1% XPC in the starter grains (Diamond V, Cedar Rapids, Iowa). * *p* < 0.1, ** *p* < 0.05 between CON and SCFP (SCFP1 & SCFP2). RL28 = Rumen liquid portions at day 28 (n = 13), RL56 = Rumen liquid portions at day 56 (n = 15), RS56 = Rumen solid contents at day 56 (n = 15), DC56= Duodenal contents at day 56 (n = 15), CC = Cecal contents at day 56 (n = 15), RC = Rectal contents at day 56 (n = 15).

Microbial species richness and diversity indices (ACE, Chao1, Shannon, Simpson) were different between sites, with higher abundance in large intestine compared to the rumen (Table 2). We found out that, SCFP can influence microbial richness and diversity in rumen, duodenum and large intestine in different ways (Figure 1a–d). ACE and OTU numbers, as indices of microbial richness, decreased in rumen (RL56, *p* = 0.09 and *p* = 0.11) and duodenum (DC56, *p* = 0.06 and *p* = 0.04), but increased in the large intestine (CC56, *p* = 0.04 and *p* = 0.04; RC56, *p* = 0.01 and *p* = 0.02) when SCFP was supplemented in the diet. Microbial diversity (evenness), Shannon (*p* = 0.08) and Simpson (*p* = 0.10) tended to decrease only in RL28. Higher level of SCFP in the diet did not exhibit further influence under the conditions of this trial. 

#### 3.2.2. Sample Clustering

**Figure 2:**
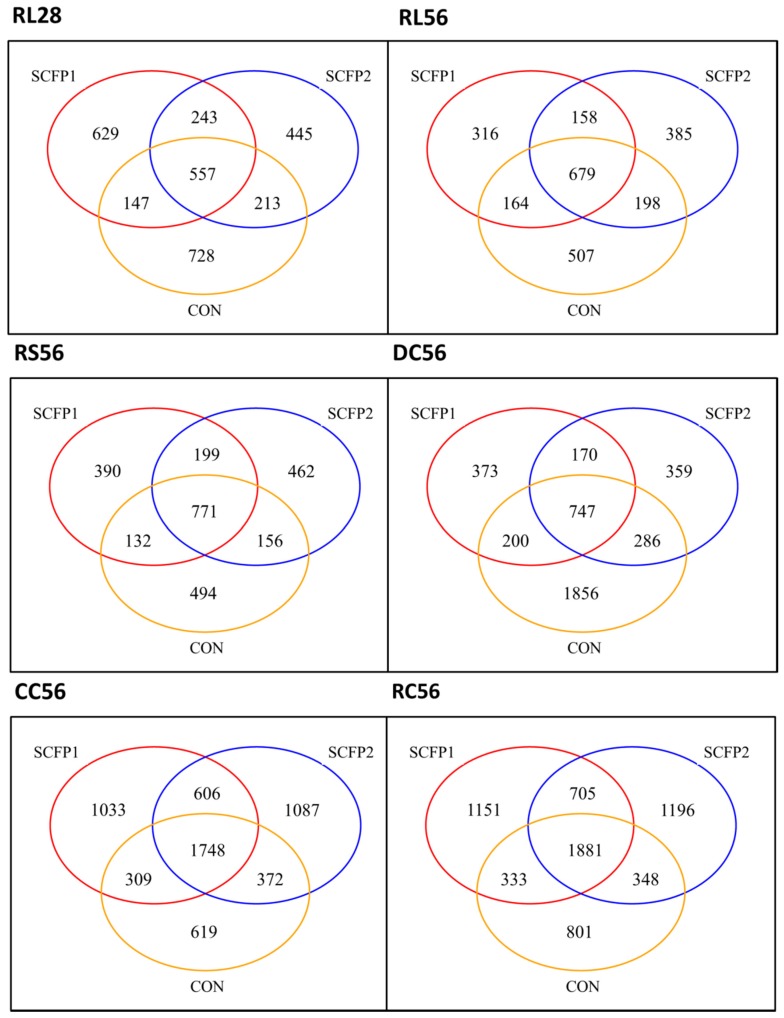
Venn plot for OTUs. The OTUs difference among CON, SCFP1 and SCFP2 in digestive sites respectively (RL28, RL56, RS56, DC56, CC56 and RC56). Singleton sequences (occurring once in only one sample) were removed before analysis.

The Venn diagrams demonstrated inconsistent overlapping patterns for each treatment in different sites (Figure 2). Along the GIT, there were different numbers of unique OTUs among treatments in RL28 (CON, 728; SCFP1, 629; and SCFP2, 445), RL56 (CON, 507; SCFP1, 316; and SCFP2, 385), DC56 (CON, 1856; SCFP1, 373; and SCFP2, 359), in CC56 (CON, 619; SCFP1, 1033; and SCFP2, 1087) and RC56 (CON, 801; SCFP1, 1151; and SCFP2, 1196). Moreover, compared to shared OTUs between CON and SCFP in RL28 (180, accounting for 6.1% of detected OTUs), CC56 (341, accounting for 5.9% of detected OTUs) and RC56 (341, accounting for 5.3% of detected OTUs), greater OTU overlap between SCFP1 and SCFP2 was exhibited in RL28 (243, accounting for 8.2% of detected OTUs), CC56 (606, accounting for 10.5% of detected OTUs), and RC56 (705, accounting for 11.0% of detected OTUs), respectively. 

**Figure 3:**
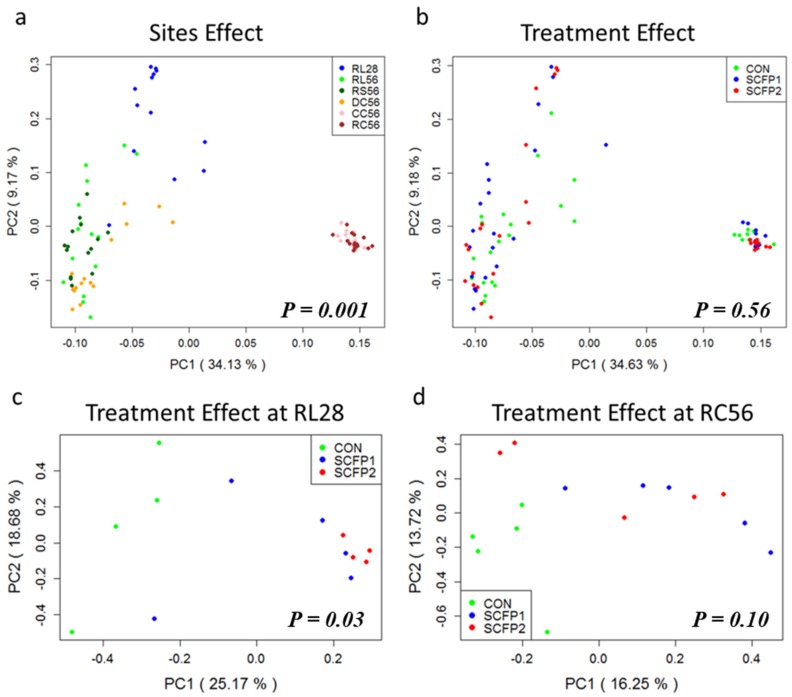
Separation of samples based on phylogenetic information by using PCoA plot, statistical comparison of microbiota was performed with ANOSIM analysis (**a**), sample clustering into variations by locations. (**b**), Sample clustering into variations by treatments. (**c**), Treatment separation in RL28. (**d**), Treatment separation in RC56.

Principal coordinate analysis (PCOA) plots clustered all samples mainly by location (Figure 3a and Figure S1, ANOSIM *p* = 0.001). Then by treatment at RL 28 (Figure 3c and Figure S1, ANOSIM *p* = 0.03) and RC56 (Figure 3d and Figure S1, ANOSIM *p* = 0.10). We did not observe strong clustering among treatments variables in other sites (Figure 3b, Figures S2 and S3, ANOSIM *p >* 0.05), suggesting that with the exception of location, supplementing SCFP has the potential to change the microbial community in early age rumen and in the rectum of the weaning calves.

#### 3.2.3. Taxonomic Characteristics

**Figure 4:**
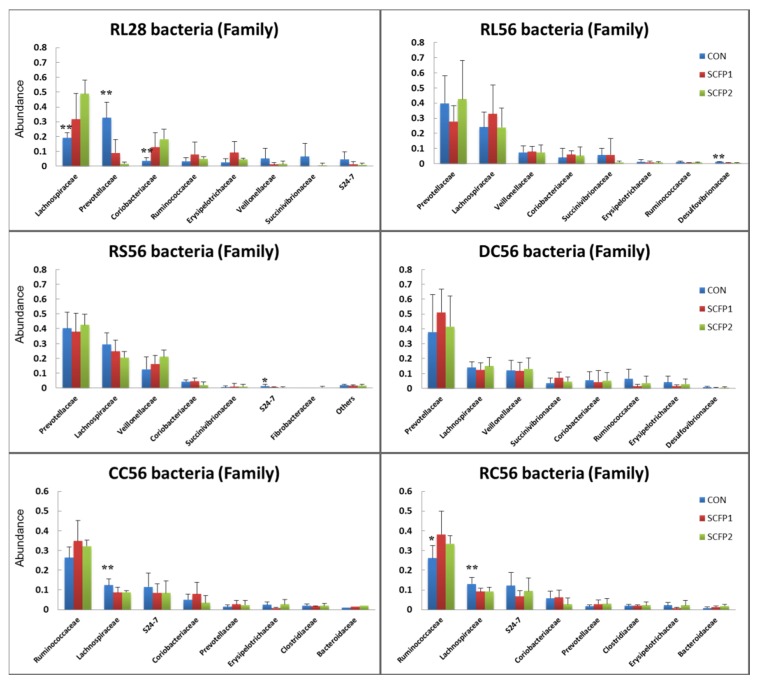
Top eight bacteria percentage in family level of total OTUs number of (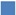) CON, (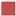) SCFP1 and (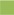) SCFP2 through GITs. CON = No SmartCare and XPC; SCFP1 = 1 g/head/d SmartCare in milk + 0.5% XPC in the starter grains; SCFP2 = 1 g/head/d SmartCare in milk + 1% XPC in the starter grains (Diamond V, Cedar Rapids, Iowa). * *p* < 0.1, ** *p* < 0.05 between CON and SCFP (SCFP1 & SCFP2). RL28 = Rumen liquid portions at day 28 (n = 13), RL56 = Rumen liquid portions at day 56 (n = 15), RS56 = Rumen solid contents at day 56 (n = 15), DC56 = Duodenal contents at day 56 (n = 15), CC = Cecal contents at day 56 (n = 15), RC = Rectal contents at day 56 (n = 15).

Overall, 25, 21 and 20 phyla and 256, 247 and 229 genera were detected for CON, SCFP1 and SCFP2, respectively. In RL28 (Table S1), seven predominant phyla (*Firmicutes*, *Bacteroidetes*, *Actinobacteria*, *Proteobacteria*, *Tenericutes*, *Cyanobacteria* and *Spirochaetes* with at least ≥ 1% relative abundance in one sample) were detected. Among these phyla, SCFP supplementation decreased relative occurrence of *Bacteroidetes* (*p* < 0.001) and *Spirochaetes* (*p* = 0.04), and increased that of *Firmicutes* (*p* = 0.02). The impact of SCFP on core family *Prevotellaceae* (*p* = 0.002) and *Lachnospiraceae* (*p* = 0.06) significantly contributed to the lower abundance of *Bacteroidetes* (*p <* 0.001) and higher abundance of *Firmicutes* (*p* = 0.02), respectively (Figure 4). On the other hand, genus *Prevotella* and *Butyrivibrio* were dominant in *Prevotellaceae* and *Lachnospiraceae*, respectively. The relatively low abundance genus, *Mogibacterium* (*p* = 0.06) increased while *Sphaerochaeta* (*p* = 0.04) decreased when supplementing SCFP in milk and starter. 

In rumen and duodenum samples at day 56, almost no significant treatment effects were observed in the RL56, RS56 and DC56 at family (Figure 4) or any other level of classification (Tables S2–S4, *p* > 0.05). However, in large intestine samples at day 56, relative occurrence of family *Lachnospiraceae* were found to decrease in RC56 (CON, 13.09%; SCFP1, 9.14%; and SCFP2, 9.31%, *p* = 0.04). Numerical increase was exhibited in family *Ruminococcus* in RC56 (CON, 26.05%; SCFP1, 38.15%; and SCFP2, 33.48%, *p* = 0.14) due to the supplementation of SCFP (Figure 4). The increase in *Ruminococcus* was likely due to genus the *Ruminococcus* and *Oscillospira* (Tables S5 and S6). 

No significant effects were observed between SCFP1 and SCFP2 for bacteria abundance in all GIT sites at day 56.

## 4. Discussion 

According to our results based on microbial alpha-diversity (Figure 1), SCFP can reduce microbial richness and evenness in rumen and increase community richness in large intestine. In other words, with supplementation of SCFP, less of different types of bacteria were detected in rumen but more types in the large intestine. A decrease in rumen community richness in SCFP groups was probably the result of the higher emergence of dominant bacteria in SCFP (Figure 4), which affected the colonization by other bacteria. The improvement in the core bacteria might change the rumen environment and as a result affect the emergence of other bacteria. For example, the *Lachnospiraceae*, butyrate-producing bacteria [28,29], increased in RL28 in SCFP (CON vs. SCFP = 19.21% vs. 40.25%, Figure 4), which concomitantly resulted in a higher butyrate concentration [18]. Li, et al. [30] demonstrated that butyrate infusion altered rumen microbial composition and lowered the numbers of OTUs. Hence, we speculate that the decrease in community richness and diversity in current study was probably induced by the increase in abundance of butyrate-producing bacteria (*Lachnospiraceae*) and the butyrate concentration in the rumen. 

*Saccharomyces cerevisiae* fermentation products are a rich in nutritional metabolites, mannan oligosaccharides and β-glucans, which can benefit various types of bacteria [15]. In a companion study, no treatment differences were observed for starter intake [18], hence we postulate that the effects hereby observed came from SCFP rather than the ration. In agreement with findings in the large intestine, previous studies revealed that inclusion SCFP considerably stimulated the microbial richness in pigs [31], which might be associated with the SCFP metabolites ability to stimulate diverse communities of microorganisms that colonize the large intestine [32]. Although microbial richness increased in current study, the impact of SCFP on microbial richness and diversity has not been studied extensively, hence future research should aim at verifying the changes observed in the present study.

It is generally believed that SCFP can modulate the structure of bacterial community in a mature rumen [33,34] and the large intestine in non-ruminants [32,35]. Similar impacts were obtained in the GIT of calves in this study (Figure 1 and Figure 4). Greater effect of SCFP was found in rumen at an early age (day 28, Figure 3) when microbiota was less stable and more heterogeneous compared to more mature age [2]. Another reason might have been related to the Smartcare feeding from day 2 to day 30, whereby both XPC and Smartcare had an effect on rumen fermentation at day 28. At day 56, similar bacterial community was seen in the rumen when only XPC were added in the starter only (Figure 3). Although no big differences existed in the rumen at day 56, we found SCFP could affect rectal microbial community drastically (Figure 3, Table S6) which could influence fiber fermentation in the hindgut of calves. As young calves have a nonfunctional rumen, it is expected for SCFP to have greater impact on the hindgut rather than the rumen. These results suggest that both rumen and hindgut should be explored when investigating the effect of SCFP or other feed additives on calves in the future. 

In line with previous bovine studies [36,37], the members of the *Prevotellaceae*, *Lachnospiraceae*, *Coriobacteriaceae,* and *Ruminococcaceae* were predominant in current study (Figure 4). This is the first time that we have demonstrated that these groups of bacteria are affected by SCFP in young calves. *Coriobacteriaceae*, belongs to pylum *Actinobacteria*, gram-positive bacteria that have been isolated from large intestine in both human and mouse, which was trended increased in SCFP groups (*p* = 0.08). They are associated with polyphenol conversion as well as bile acid and hepatic lipid metabolism [38]. In cattle, they represent up to 3% of mature rumen bacteria [39], which was similar to the current study. However, their role and function in rumen is largely unknown and need further investigation to explain the presence of higher *Coriobacteriaceae* in the present study when SCFP was supplemented. 

*Prevotellaceae*, belongs to phylum *Bacteroidetes*, gram-negative bacteria with the ability to utilize various sugars [40] and are believed to play a crucial role in starch degradation [41]. Members of the *Ruminococcaceae* and *Lachnospiraceae* families are largely fibrolytic bacteria and belong to the phylum *Firmicutes* [36]. The lower emergence of *Prevotellaceae* and higher emergence of *Lachnospiraceae* suggest that SCFP might change the fermentation type and stimulate the degradation of recalcitrant fiber substrates in pre-mature rumen of calves. 

In the large intestine, as the essential types of bacteria that produce SCFAs, *Lachnospiraceae* and *Ruminococcaceae* are significantly depleted in diarrheic patients [42]. The abundance of *Ruminococcaceae* increased (CON vs. SCFP = 26.23% vs. 34.66%) while that of *Lachnospiraceae* decreased (CON vs. SCFP = 12.72% vs. 8.95%) in SCFP groups compared to CON. A recent comparitive genomic study on the carbohydrate-active enzymes, transporters and metabolic pathways between *Lachnospiraceae* and *Ruminococcaceae*, revealed that although these two members were specialized in the degradation of plant material, *Lachnospiraceae* were richer in starch-degrading alpha-glucosidases and phosphorylases genes while *Ruminococcaceae* were rich in cellulase genes and endo-1, 4-beta-xylanases [43]. Therefore, we speculate that the higher abundance of *Ruminococcaceae* in SCFP groups in CC56 and RC56 might stimulate the degradation of cellulose, which has not or been poorly digested in the rumen with less than 1% of *Ruminococcaceae* in each group (Table S2). On the other hand, the lower *Lachnospiraceae* in SCFP groups in large intestine might be because of the higher *Lachnospiraceae* (dominated by genus *Butyrivibrio*) exhibited in rumen, where most of the substrates for *Lachnospiraceae* had already been largely utilized, resulting in less residues arriving in large intestine when SCFP was supplemented. In a companion paper [18], we found SCFP groups exhibited a higher butyrate production in the rumen. The higher butyrate was probably produced by butyrate-producing anaerobic bacteria, genus *Butyrivibrio* [28], which is further proof that specific substrates were utilized by higher *Lachnospiraceae* in SCFP groups in rumen. Although our data clearly showed differences in the microbiota structure as a result of dietary supplementation with SCFP, deciphering these changes at the family or other levels and relating these changes to ecological function remain a formidable challenge and need further investigation. 

## 5. Conclusions

This study demonstrated that the bacterial composition of pre-weaned calves varied by the dietary supplementation of SCFP. *Saccharomyces cerevisiae* fermentation products have an ability to increase the species richness in large intestine. It also changed the bacterial composition and stimulated the fiber digesting bacteria in rumen at early age (RL28) and later in large intestine (RC56). Furthermore, SCFP are more likely to change the bacterial composition in hindgut rather than rumen at day 56, as SCFP supplemented calves increased the abundance *Ruminococcaceae* in large intestine. When calves were supplemented with SCFP at a higher rate in the starter (1% vs. 0.5%) no further changes were observed in bacterial community.

## Figures and Tables

**Table 1:** Diversity estimation of the 16S rRNA gene libraries of all 88 samples.

Sites/Groups ^a^	N ^b^	Average Reads	Average OTUs ^c^
RL28			
CON	4	160,855	1101
SCFP1	5	155,655	1042
SCFP2	4	133,215	891
RL56			
CON	5	158,254	1059
SCFP1	5	177,990	928
SCFP2	5	161,029	962
RS56			
CON	5	144,101	1120
SCFP1	5	119,776	1075
SCFP2	5	141,623	1153
DC56			
CON	5	156,828	1877
SCFP1	5	118,575	1045
SCFP2	5	161,956	1053
CC56			
CON	5	183,365	2084
SCFP1	5	185,415	2460
SCFP2	5	178,122	2561
RC56			
CON	5	184,172	2338
SCFP1	5	203,071	2829
SCFP2	5	196,179	2827

^a^ RL28 = Rumen liquid portions at day 28, RL56 = Rumen liquid portions at day 56, RS56 = Rumen solid contents at day 56, DC56 = Duodenal contents at day 56, CC56 = Cecal contents at day 56, RC56 = Rectal contents at day 56, RN56 = Rumen contents without fluid at day 56. CON = No SmartCare and XPC; SCFP1 = 1 g/hd/d SmartCare + 0.5%XPC; SCFP2 = 1 g//hd/d SmartCare + 1%XPC. ^b^ N = the number of calves was used. ^c^ OTUs = Operational Taxonomic Units.

**Table 2:** The effect of gastrointestinal sites on the estimates of microbial richness and diversity in calves.

Item/Index ^1^	N ^2^	RL56	RS56	DC56	CC56	RC56	SEM
		Mean ^3^					
ACE	15	2038.2 ^a^	2360.9 ^ab^	2685.3 ^b^	4358.6 ^c^	4893.5 ^c^	156.6
Chao1	15	1998.9 ^a^	2293.6 ^a^	2537.7 ^a^	4271.7 ^b^	4810.1 ^b^	153.4
Shannon	15	4.88 ^a^	5.33 ^a^	5.28 ^a^	7.18 ^b^	7.35 ^b^	0.16
Simpson	15	0.90 ^a^	0.93 ^ab^	0.92 ^a^	0.97 ^b^	0.97 ^b^	0.01
OTUs	15	982.9 ^a^	1116.0 ^a^	1325.0 ^a^	2363.5 ^b^	2664.7 ^b^	94.6

^a,b,c^ Means within a row not bearing a common superscript differ (*p* < 0.05). ^1^ CON = No SmartCare or XPC; SCFP1 = 1 g/head/d SmartCare in milk + 0.5% XPC in the starter grains; SCFP2 = 1 g/head/d SmartCare in milk + 1% XPC in the starter grains (Diamond V, Cedar Rapids, Iowa); Total means CON+SCFP1+SCPF2. ^2^ N = the number of samples was used in each site. ^3^ Mean =least square mean, RL56 = Rumen liquid portions at day 56, RS56 = Rumen solid contents at day 56, DC56= Duodenal contents at day 56, CC = Caecal contents at day 56, RC = Rectal contents at day 56.

## Supplementary Materials

The following are available online at http://www.mdpi.com/2076-2615/9/1/4/s1, Figure S1: ANOSIM analysis were performed at sites (a) and treatments in GITs (b), RL28. (c), and RC56 (d), Figure S2: ANOSIM analysis were performed among treatments in RL56 (a), RS56 (b), DC56 (c) and CC56 (d), Figure S3: Separation of samples based on phylogenetic information using PCoA plot. (a), treatment separation in RL56. (b), treatment separation in RS56. (c), treatment separation in DC56. (d), treatment separation in CC56, Table S1: The effect of SCFP on Bacterial abundance in each level in rumen liquid fraction sampled on day 28 (RL28), Table S2: The effect of SCFP on Bacterial abundance in each level in rumen liquid fraction sampled on day 56 (RL56), Table S3: The effect of SCFP on Bacterial abundance in each level in rumen solid fraction sampled on day 56 (RS56), Table S4: The effect of SCFP on Bacterial abundance in each level in duodenal content sampled on day 56 (DC56), Table S5: The effect of SCFP on Bacterial abundance in each level in cecal content sampled on day 56 (CC56); Table S6: The effect of SCFP on Bacterial abundance in each level in rectal content sampled on day 56 (RC56).
