# Supplementary material for: Effects of Saccharomyces Cerevisiae Fermentation Products on the Microbial Community throughout the Gastrointestinal Tract of Calves
Source: Animals (Basel). 2018 Dec 21;9(1):4. doi: 10.3390/ani9010004 (PMC6356610; doi:10.3390/ani9010004)
Supplement: Supplementary file 1 [file animals-09-00004-s001.pdf]

Table S1. The effect of SCFP on Bacterial abundance in each level in rumen liquid fraction sampled on day 28 (RL28).

Item/Index[1]	CON	SCFP1	SCFP2	SEM	*P* value	
**Phylum** ‖ Order │ Family ┆ Genus	Mean (%)			(%)	CON vs SCFP	SCFP 1 vs 2
Actinobacteria	5.78	13.91	18.87	4.76	0.18	0.90
‖ *Coriobacteriales*	3.74	12.53	18.35	4.10	0.08	0.68
│ *Coriobacteriaceae*	3.74	12.80	18.35	4.10	0.08	0.68
‖ *Bifidobacteriales*	2.00	1.16	0.41	1.26	0.80	1.00
│ *Bifidobacteriaceae*	2.00	0.93	0.41	1.26	0.80	1.00
┆ *Bifidobacterium*	1.25	0.80	0.41	0.87	1.00	1.00
Bacteroidetes	42.90	17.36	5.57	6.06	<0.001	0.36
‖ *Bacteroidales*	42.84	19.92	5.50	6.03	0.004	0.36
│ *Prevotellaceae*	32.72	9.08	1.67	4.57	0.002	0.50
┆ *Prevotella*	32.72	9.08	1.67	4.57	0.002	0.50
│ *[Paraprevotellaceae]*	0.80	0.18	0.07	0.28	0.16	1.00
│ *S24-7*	4.68	1.32	1.12	1.76	0.26	1.00
Cyanobacteria	0.27	0.61	0.63	0.18	0.26	1.00
Firmicutes	42.63	65.18	69.22	6.94	0.02	1.00
‖ *Clostridiales*	39.89	54.12	64.47	5.32	0.02	0.52
│ *Lachnospiraceae*	19.22	31.69	48.81	6.95	0.06	0.20
┆ *Butyrivibrio*	6.74	22.97	45.14	6.93	0.01	0.08
┆ *Shuttleworthia*	4.59	0.19	0.13	1.32	0.38	1.00
┆ *Roseburia*	0.35	0.11	0.07	0.15	0.40	1.00
│ *Ruminococcaceae*	3.33	8.05	5.01	3.10	0.81	0.96
┆ *Ruminococcus*	0.85	1.29	1.17	0.59	1.00	1.00
┆ *Oscillospira*	0.43	1.39	0.39	0.69	1.00	0.62
│ *Veillonellaceae*	5.15	1.30	1.49	2.31	0.42	1.00
┆ *Dialister*	1.53	0.25	0.77	0.86	0.70	1.00
┆ *Megasphaera*	2.03	0.16	0.06	1.03	0.30	1.00
┆ *Succiniclasticum*	0.23	0.29	0.09	0.14	1.00	0.66
│ *Mogibacteriaceae*	0.10	0.34	0.75	0.14	0.06	0.12
┆ *Mogibacterium*	0.02	0.24	0.60	0.13	0.06	0.12
‖ *Erysipelotrichales*	2.69	8.22	4.52	2.85	0.50	0.50
│ *Erysipelotrichaceae*	2.69	9.17	4.52	2.85	0.50	0.50
┆ *Bulleidia*	2.51	8.85	4.20	2.85	0.52	0.50

Proteobacteria	7.04	1.49	2.01	2.84	0.30	1.00
Aeromonadales	6.46	0.06	0.80	2.84	0.22	1.00
Succinivibrionaceae	6.46	0.06	0.80	2.84	0.22	1.00
Succinivibrio	6.43	0.04	0.73	2.83	0.22	1.00
Pseudomonadales	0.09	0.23	0.43	0.14	0.42	0.58
Moraxellaceae	0.08	0.21	0.43	0.14	0.42	0.56
Spirochaetes	0.60	0.02	0.02	0.17	0.04	1.00
Sphaerochaetales	0.60	0.02	0.02	0.17	0.04	1.00
Sphaerochaetaceae	0.60	0.02	0.02	0.17	0.04	1.00
Sphaerochaeta	0.60	0.02	0.02	0.17	0.04	1.00
Tenericutes	0.37	0.56	3.58	1.23	0.56	0.20
RF39	0.37	0.61	3.58	1.23	0.56	0.20

[1]Only predominant bacteria (abundance ≥ 1% in at least one sample) were compared in Phylum, Order, Family and genus level. 13 samples were used in RL28 analysis (CON: 4 samples, SCFP1: 5 samples, SCFP2: 4 samples).

Table S2. The effect of SCFP on Bacterial abundance in each level in rumen liquid fraction sampled on day 56 (RL56).

Item/Index[1] Phylum / Order / Family / Genus	CON	SCFP1	SCFP2	SEM (%)	*P* value CON vs SCFP	*P* value SCFP 1 vs 2
	Mean (%)					
Actinobacteria	4.05	6.15	5.40	2.56	1.00	1.00
Coriobacteriales	4.03	6.00	5.32	2.55	1.00	1.00
Coriobacteriaceae	4.03	6.00	5.32	2.55	1.00	1.00
Bacteroidetes	44.73	29.34	43.96	9.77	1.00	0.62
Bacteroidales	44.73	29.34	43.96	9.77	1.00	0.62
Prevotellaceae	43.83	27.81	42.56	9.51	1.00	0.48
Prevotella	43.83	27.81	36.56	6.93	0.40	0.78
S24-7	0.30	0.14	0.30	0.15	1.00	0.92
p-2534-18B5	0.49	0.12	0.30	0.14	0.24	0.74
Cyanobacteria	0.33	0.37	0.22	0.14	1.00	0.90
YS2	0.27	0.32	0.10	0.13	1.00	0.58
Fibrobacteres	0.01	0.00	0.24	0.14	1.00	0.48
Fibrobacterales	0.01	0.00	0.24	0.14	1.00	0.48
Fibrobacteraceae	0.01	0.00	0.24	0.14	1.00	0.48
Fibrobacter	0.01	0.00	0.24	0.14	1.00	0.48
Firmicutes	43.27	57.20	48.06	9.69	0.90	1.00
Clostridiales	41.87	56.26	47.28	9.37	0.82	1.00
Lachnospiraceae	24.19	32.79	23.87	7.22	1.00	0.98
Butyrivibrio	10.74	20.93	20.84	4.62	0.20	1.00
Shuttleworthia	0.47	0.67	2.90	1.26	0.82	0.46
Veillonellaceae	7.47	7.92	7.34	2.12	1.00	1.00
Dialister	2.31	2.93	2.06	1.00	1.00	1.00
Megasphaera	0.14	0.85	0.25	0.23	0.34	0.20
Succiniclasticum	1.26	0.50	0.60	0.46	0.46	1.00
Ruminococcaceae	1.09	0.50	0.72	0.22	0.22	0.98
Erysipelotrichales	1.26	0.89	0.76	0.46	0.90	1.00
Erysipelotrichaceae	1.26	0.89	0.76	0.46	0.90	1.00
Bulleidia	0.61	0.61	0.56	0.24	1.00	1.00
Sharpea	0.62	0.22	0.12	0.32	0.56	1.00
Proteobacteria	6.80	6.33	1.52	3.31	0.98	0.64
Aeromonadales	5.57	5.81	0.96	3.38	1.00	0.66
Succinivibrionaceae	5.57	5.80	0.96	3.38	1.00	0.66
Succinivibrio	1.53	3.51	0.38	2.03	1.00	0.60

Desulfovibrionales	1.07	0.43	0.50	0.14	0.02	1.00
Desulfovibrionaceae	1.07	0.43	0.50	0.14	0.02	1.00
Desulfovibrio	1.07	0.43	0.47	0.15	0.02	1.00
Tenericutes	0.46	0.08	0.16	0.23	0.50	1.00
RF39	0.46	0.08	0.16	0.23	0.50	1.00

[1]Only predominant bacteria (abundance ≥ 1% in at least one sample) were compared in Phylum, Order, Family and genus level. 15 samples were used in RL56 analysis (CON: 5 samples, SCFP1: 5 samples, SCFP2: 5 samples).

Table S3. The effect of SCFP on Bacterial abundance in each level in rumen solid fraction sampled on day 56 (RS56).

Item/Index[1] Phylum ‖ Order │ Family ┆ Genus	CON	SCFP1	SCFP2	SEM (%)	P value: CON vs SCFP	P value: SCFP 1 vs 2
	Mean (%)					
Actinobacteria	4.27	4.63	2.10	0.89	0.84	0.14
‖ *Coriobacteriales*	4.27	4.60	2.09	0.89	0.84	0.14
│ *Coriobacteriaceae*	4.27	4.60	2.09	0.89	0.82	0.14
Bacteroidetes	44.26	40.90	47.26	5.01	1.00	0.74
‖ *Bacteroidales*	44.26	40.89	47.26	5.01	1.00	0.78
│ *Prevotellaceae*	40.29	38.00	42.59	5.17	1.00	1.00
┆ *Prevotella*	40.29	38.00	42.59	5.17	1.00	1.00
│ *S24-7*	1.23	0.47	0.46	0.35	0.20	1.00
Fibrobacteres	0.00	0.00	0.30	0.17	0.98	0.48
‖ *Fibrobacterales*	0.00	0.00	0.30	0.17	0.98	0.48
│ *Fibrobacteraceae*	0.00	0.00	0.30	0.17	0.98	0.48
┆ *Fibrobacter*	0.00	0.00	0.30	0.17	0.98	0.48
Firmicutes	50.02	52.52	48.54	4.93	1.00	1.00
‖ *Clostridiales*	49.41	52.01	48.06	4.92	1.00	1.00
│ *Lachnospiraceae*	29.43	24.68	20.47	3.35	0.24	1.00
┆ *Butyrivibrio*	19.49	14.45	12.44	3.16	0.28	1.00
┆ *Succiniclasticum*	3.75	2.30	3.24	1.51	1.00	1.00
│ *Veillonellaceae*	12.59	16.34	21.10	3.17	0.28	0.62
┆ *Dialister*	6.48	5.06	6.43	2.01	1.00	1.00
┆ *Acidaminococcus*	0.50	0.76	0.86	0.22	0.54	1.00
┆ *Megasphaera*	0.13	0.69	0.34	0.24	0.44	0.68
┆ *Shuttleworthia*	0.12	2.03	0.10	0.77	0.66	0.20
┆ *Mitsuokella*	0.09	0.29	0.20	0.11	0.54	1.00
Proteobacteria	0.84	1.37	1.22	0.68	1.00	1.00
‖ *Aeromonadales*	0.51	1.09	1.03	0.68	1.00	1.00
│ *Succinivibrionaceae*	0.50	1.09	1.02	0.68	1.00	1.00
┆ *Succinivibrio*	0.16	0.95	0.72	0.62	1.00	1.00

[1]Only predominant bacteria (abundance ≥ 1% in at least one sample) were compared in Phylum, Order, Family and genus level. 15 samples were used in RS56 analysis (CON: 5 samples, SCFP1: 5 samples, SCFP2: 5 samples).

Table S4. The effect of SCFP on Bacterial abundance in each level in duodenal content sampled on day 56 (DC56).

Item/Index[1]	CON	SCFP1	SCFP2	SEM (%)	*P* value	
**Phylum** / ║ Order / │ Family / ┆ Genus	Mean (%)				CON vs SCFP	SCFP 1 vs 2
Actinobacteria	5.88	4.34	5.29	3.20	1.00	1.00
║ *Coriobacteriales*	5.66	4.24	5.16	3.14	1.00	1.00
│ *Coriobacteriaceae*	5.66	4.24	5.16	3.14	1.00	1.00
Bacteroidetes	39.27	51.82	42.45	10.30	1.00	1.00
║ *Bacteroidales*	39.25	51.82	42.44	10.31	1.00	1.00
│ *Prevotellaceae*	37.85	51.13	41.53	10.40	1.00	1.00
┆ *Prevotella*	37.85	51.13	41.53	10.40	1.00	1.00
│ *S24-7*	0.76	0.17	0.17	0.17	0.02	1.00
Cyanobacteria	0.53	0.68	0.70	0.18	1.00	1.00
║ *YS2*	0.38	0.48	0.30	0.15	1.00	0.84
║ *Streptophyta*	0.15	0.20	0.40	0.10	0.50	0.32
Firmicutes	47.86	34.74	44.94	8.37	0.90	0.82
║ *Clostridiales*	43.50	33.27	41.92	7.22	1.00	0.82
│ *Lachnospiraceae*	14.25	12.41	14.96	2.43	1.00	0.94
┆ *Butyrivibrio*	5.55	5.43	6.05	1.49	1.00	1.00
┆ *Shuttleworthia*	0.97	3.88	3.87	2.38	0.68	1.00
│ *Veillonellaceae*	12.26	11.71	13.13	3.28	1.00	1.00
┆ *Dialister*	5.12	4.96	3.86	1.78	1.00	1.00
┆ *Megasphaera*	1.59	2.02	4.45	2.19	1.00	0.90
┆ *Succiniclasticum*	1.59	0.57	0.32	0.60	0.30	1.00
┆ *Acidaminococcus*	0.98	0.57	0.73	0.25	0.62	1.00
┆ *Mitsuokella*	0.28	0.27	0.38	0.17	1.00	1.00
│ *Ruminococcaceae*	6.61	1.41	3.63	2.28	0.34	1.00
┆ *Ruminococcus*	0.51	0.05	0.14	0.17	0.14	1.00
┆ *Oscillospira*	0.68	0.10	0.21	0.23	0.16	1.00
│ *Mogibacteriaceae*	0.62	0.12	0.31	0.25	0.42	1.00
║ *Erysipelotrichales*	4.06	1.42	2.95	1.54	0.68	1.00
│ *Erysipelotrichaceae*	4.06	1.42	2.95	1.54	0.68	1.00
┆ *Bulleidia*	1.65	0.74	2.20	1.03	1.00	0.68
┆ *Sharpea*	2.23	0.61	0.57	0.77	0.22	1.00
Proteobacteria	5.62	7.94	5.51	1.61	1.00	0.62
║ *Aeromonadales*	3.67	7.13	4.68	1.68	0.60	0.64
│ *Succinivibrionaceae*	3.66	7.13	4.67	1.68	0.60	0.64
┆ *Succinivibrio*	0.72	2.51	0.83	0.78	0.68	0.32

‖ *Desulfovibrionales*	0.77	0.39	0.49	0.23	0.54	1.00
│ *Desulfovibrionaceae*	0.77	0.39	0.49	0.23	0.54	1.00
┆ *Desulfovibrio*	0.75	0.39	0.48	0.23	0.60	1.00
‖ *Pseudomonadales*	0.38	0.05	0.03	0.10	0.04	1.00
Tenericutes	0.17	0.09	0.69	0.39	1.00	0.60
‖ *RF39*	0.16	0.09	0.68	0.39	1.00	0.60
Acidobacteria	0.001	0	0	<0.001	0.08	1.00
Chloroflexi	0.004	0.001	0.001	0.001	0.16	1.00
Deferribacteres	0.002	0	0	<0.001	0.20	1.00
Elusimicrobia	0.002	0.000	0.001	<0.001	0.24	1.00
Nitrospirae	0.004	0	0	0.00	0.06	1.00
Spirochaetes	0.02	0.01	0.01	0.004	0.10	1.00
TM7	0.017	0.002	0.004	0.01	0.20	1.00

[1]Only predominant bacteria (abundance ≥ 1% in at least one sample) were compared in Phylum, Order, Family and genus level. 15 samples were used in DC56 analysis (CON: 5 samples, SCFP1: 5 samples, SCFP2: 5 samples).

Table S5. The effect of SCFP on Bacterial abundance in each level in cecal content sampled on day 56 (CC56).

Item/Index[1]	Level	CON	SCFP1	SCFP2	SEM	*P* value	
Phylum / Order / Family / Genus		Mean (%)			(%)	CON vs SCFP	SCFP 1 vs 2
Actinobacteria	Phylum	5.14	8.05	3.55	2.17	1.00	0.34
Coriobacteriales	Order	5.04	7.99	3.50	2.17	1.00	0.34
Coriobacteriaceae	Family	5.04	7.99	3.50	2.17	1.00	1.00
Bacteroidetes	Phylum	21.43	22.21	23.31	3.92	1.00	1.00
Bacteroidales	Order	21.43	22.21	23.31	3.92	1.00	1.00
S24-7	Family	11.52	8.51	8.36	2.98	0.82	1.00
Prevotellaceae	Family	1.51	2.66	2.36	0.91	0.78	1.00
Prevotella	Genus	1.45	2.53	2.27	0.84	0.76	1.00
Bacteroidaceae	Family	1.03	1.39	1.88	0.42	0.52	0.84
Bacteroides	Genus	1.02	1.38	1.84	0.41	0.54	0.90
Porphyromonadaceae	Family	0.77	0.84	1.04	0.24	1.00	1.00
Parabacteroides	Genus	0.77	0.84	1.04	0.24	1.00	1.00
[Odoribacteraceae]	Family	0.22	0.28	0.45	0.11	0.62	0.58
Odoribacter	Genus	0.19	0.20	0.42	0.11	0.80	0.34
[Paraprevotellaceae]	Family	0.31	0.60	0.53	0.13	0.30	1.00
Firmicutes	Phylum	65.25	64.16	66.63	3.98	1.00	1.00
Clostridiales	Order	61.84	63.26	63.97	3.66	1.00	1.00
Ruminococcaceae	Family	26.41	34.76	32.25	3.50	0.24	1.00
Ruminococcus	Genus	4.15	12.32	7.85	3.26	0.32	0.70
Oscillospira	Genus	0.57	1.02	1.23	0.17	0.04	0.80
Faecalibacterium	Genus	0.67	0.52	1.26	0.63	1.00	0.84
Lachnospiraceae	Family	12.35	8.59	8.75	1.30	0.08	1.00
Dorea	Genus	1.90	1.98	1.66	0.78	1.00	1.00
Blautia	Genus	1.84	0.76	0.79	0.44	0.14	01.00
Coprococcus	Genus	0.25	0.63	0.45	0.23	0.60	1.00
Clostridiaceae	Family	2.10	1.72	1.88	0.48	1.00	1.00
Clostridium	Genus	1.23	0.77	0.87	0.29	0.54	1.00
Mogibacteriaceae	Family	1.34	0.43	0.46	0.29	0.06	1.00
Mogibacterium	Genus	0.75	0.22	0.24	0.14	0.02	1.00
Veillonellaceae	Family	0.77	0.31	0.42	0.42	0.88	1.00
Phascolarctobacterium	Genus	0.38	0.21	0.40	0.24	1.00	1.00
Anaerovibrio	Genus	0.25	0.05	0.01	0.13	0.38	1.00
Erysipelotrichales	Order	2.39	0.86	2.66	0.86	1.00	0.34
Erysipelotrichaceae	Family	2.39	0.86	2.66	0.86	1.00	0.34
p-75-a5	Genus	1.46	0.54	2.19	0.71	1.00	0.26

‖ *Lactobacillales*	0.99	0.01	0.001	0.55	0.34	1.00
│ *Streptococcaceae*	0.63	0.004	<0.001	0.35	0.36	1.00
┆ *Streptococcus*	0.62	0.004	<0.001	0.35	0.36	1.00
│ *Lactobacillaceae*	0.37	0.01	0.001	0.19	0.30	1.00
┆ *Lactobacillus*	0.37	0.005	0.001	0.19	0.30	1.00
Tenericutes	7.43	4.98	5.59	1.27	0.38	1.00
‖ *RF39*	7.38	4.90	5.51	1.27	0.38	1.00
Chloroflexi	0	0.001	0.001	<0.001	0.26	1.00
Elusimicrobia	0.01	0.01	0.00	0.003	1.00	0.14
Planctomycetes	0.004	0.001	0	0.002	0.20	1.00
Spirochaetes	0.03	0.01	0.04	0.01	1.00	0.50

[1]Only predominant bacteria (abundance ≥ 1% in at least one sample) were compared in Phylum, Order, Family and genus level. 15 samples were used in CC56 analysis (CON: 5 samples, SCFP1: 5 samples, SCFP2: 5 samples).

Table S6. The effect of SCFP on Bacterial abundance in each level in rectal content sampled on day 56 (RC56).

Item/Index[1] Phylum ‖ Order │ Family ┆ Genus	CON	SCFP1	SCFP2	SEM (%)	P value: CON vs SCFP	P value: SCFP 1 vs 2
	Mean (%)					
Actinobacteria	5.98	6.32	2.83	1.73	1.00	0.36
‖ *Coriobacteriales*	5.87	6.26	2.79	1.72	1.00	0.36
│ *Coriobacteriaceae*	5.87	6.26	2.79	1.72	1.00	0.36
Bacteroidetes	22.75	20.99	26.23	4.50	1.00	0.86
‖ *Bacteroidales*	22.74	20.99	26.22	4.50	1.00	0.86
│ *S24-7*	12.21	6.86	9.49	2.86	0.54	1.00
│ *Prevotellaceae*	1.71	2.86	2.95	1.01	0.70	0.95
┆ *Prevotella*	1.63	2.69	2.83	0.94	0.70	1.00
│ *Bacteroidaceae*	0.83	1.31	1.81	0.35	0.24	0.68
┆ *Bacteroides*	0.83	1.31	1.78	0.35	0.24	0.72
│ *Porphyromonadaceae*	0.76	0.79	0.96	0.20	1.00	1.00
┆ *Parabacteroides*	0.76	0.79	0.96	0.20	1.00	1.00
│ *[Paraprevotellaceae]*	0.32	0.64	0.52	0.13	0.24	1.00
Firmicutes	63.57	67.58	65.88	4.29	1.00	1.00
‖ *Clostridiales*	60.27	66.78	63.65	4.06	1.00	1.00
│ *Ruminococcaceae*	26.05	38.15	33.48	4.06	0.14	0.86
┆ *Ruminococcus*	3.46	12.60	8.52	2.97	0.14	0.70
┆ *Oscillospira*	0.59	1.23	1.29	0.17	0.02	1.00
┆ *Faecalibacterium*	0.27	0.46	1.40	0.63	0.82	0.62
│ *Lachnospiraceae*	13.09	9.14	9.31	1.22	0.04	1.00
┆ *Dorea*	1.71	2.36	1.63	0.77	1.00	1.00
┆ *Blautia*	1.25	0.80	0.79	0.29	0.48	1.00
┆ *Coprococcus*	0.37	0.54	0.57	0.16	0.68	1.00
│ *Clostridiaceae*	2.00	2.00	2.16	0.58	1.00	1.00
┆ *Clostridium*	0.95	0.72	0.92	0.24	1.00	1.00
│ *Mogibacteriaceae*	1.31	0.45	0.35	0.24	0.02	1.00
┆ *Mogibacterium*	0.67	0.19	0.13	0.10	0.002	1.00
│ *Veillonellaceae*	0.07	0.29	0.45	0.17	0.32	0.98
‖ *Erysipelotrichales*	2.27	0.74	2.22	0.80	0.88	0.44
│ *Erysipelotrichaceae*	2.27	0.74	2.22	0.80	0.88	0.44
┆ *p-75-a5*	1.38	0.44	1.81	0.67	1.00	0.34
‖ *Lactobacillales*	1.00	0.02	0.003	0.52	0.30	1.00
│ *Streptococcaceae*	0.61	0.01	0.001	0.33	0.30	1.00
┆ *Streptococcus*	0.61	0.005	0.001	0.32	0.30	1.00

Lactobacillaceae	0.38	0.01	0.002	0.19	0.28	1.00
Lactobacillus	0.38	0.01	0.002	0.19	0.28	1.00
Tenericutes	6.85	4.48	4.18	1.18	0.22	1.00
RF39	6.80	4.41	4.11	1.18	0.20	1.00
Cyanobacteria	0.21	0.15	0.08	0.04	0.16	0.50
Elusimicrobia	0.01	0.02	0.003	0.002	0.88	0.02
Planctomycetes	0.002	0.001	0	<0.001	0.42	0.48
Proteobacteria	0.31	0.14	0.20	0.10	0.56	1.00
Spirochaetes	0.03	0.01	0.05	0.02	1.00	0.14
TM7	0.001	0	0	<0.001	0.08	1.00

[1]Only predominant bacteria (abundance ≥ 1% in at least one sample) were compared in Phylum, Order, Family and genus level. 15 samples were used in RC56 analysis (CON: 5 samples, SCFP1: 5 samples, SCFP2: 5 samples).

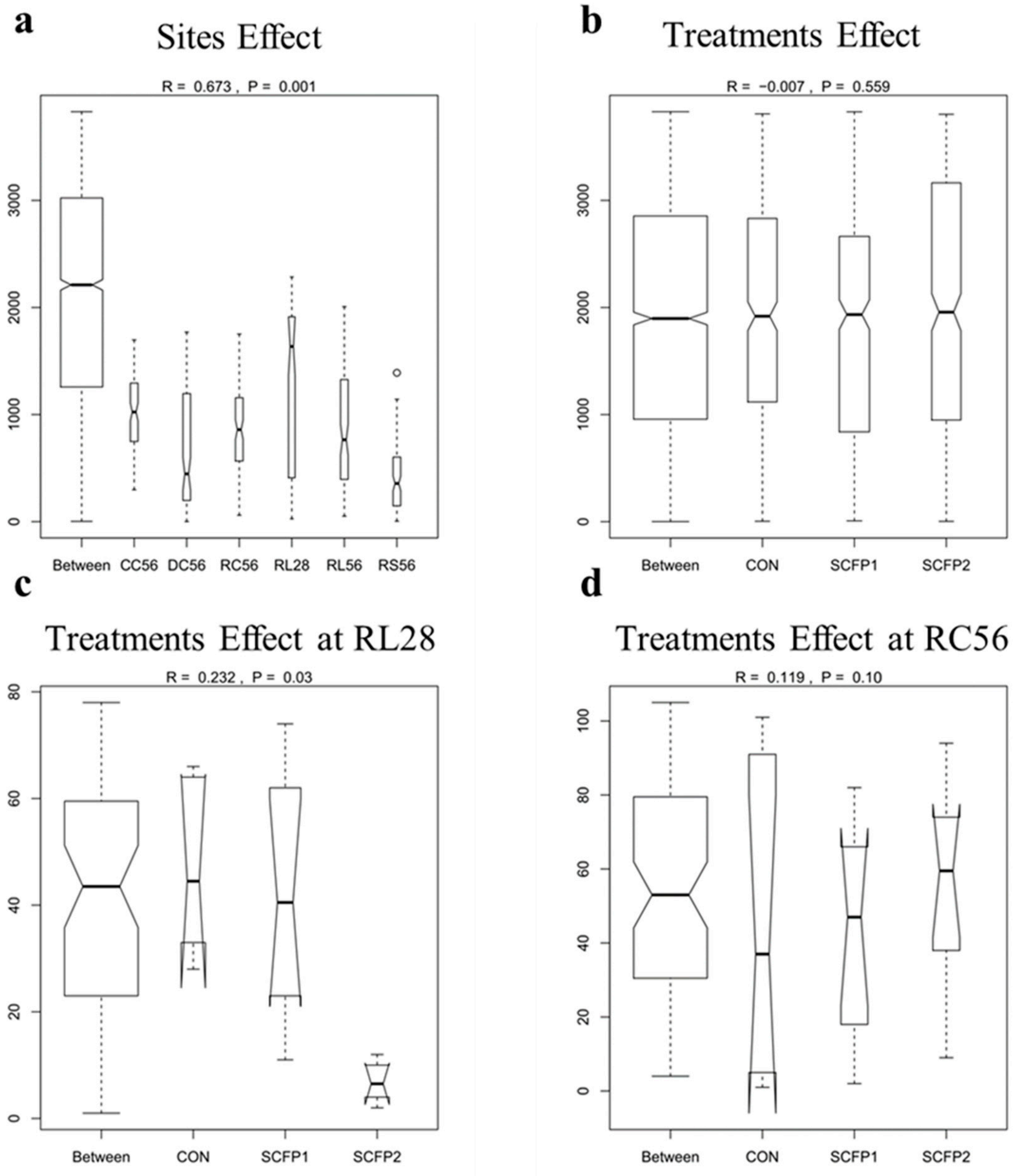

Figure S1. ANOSIM analysis were performed at sites (a) and treatments in GITs (b), RL28. (c), and RC56 (d).

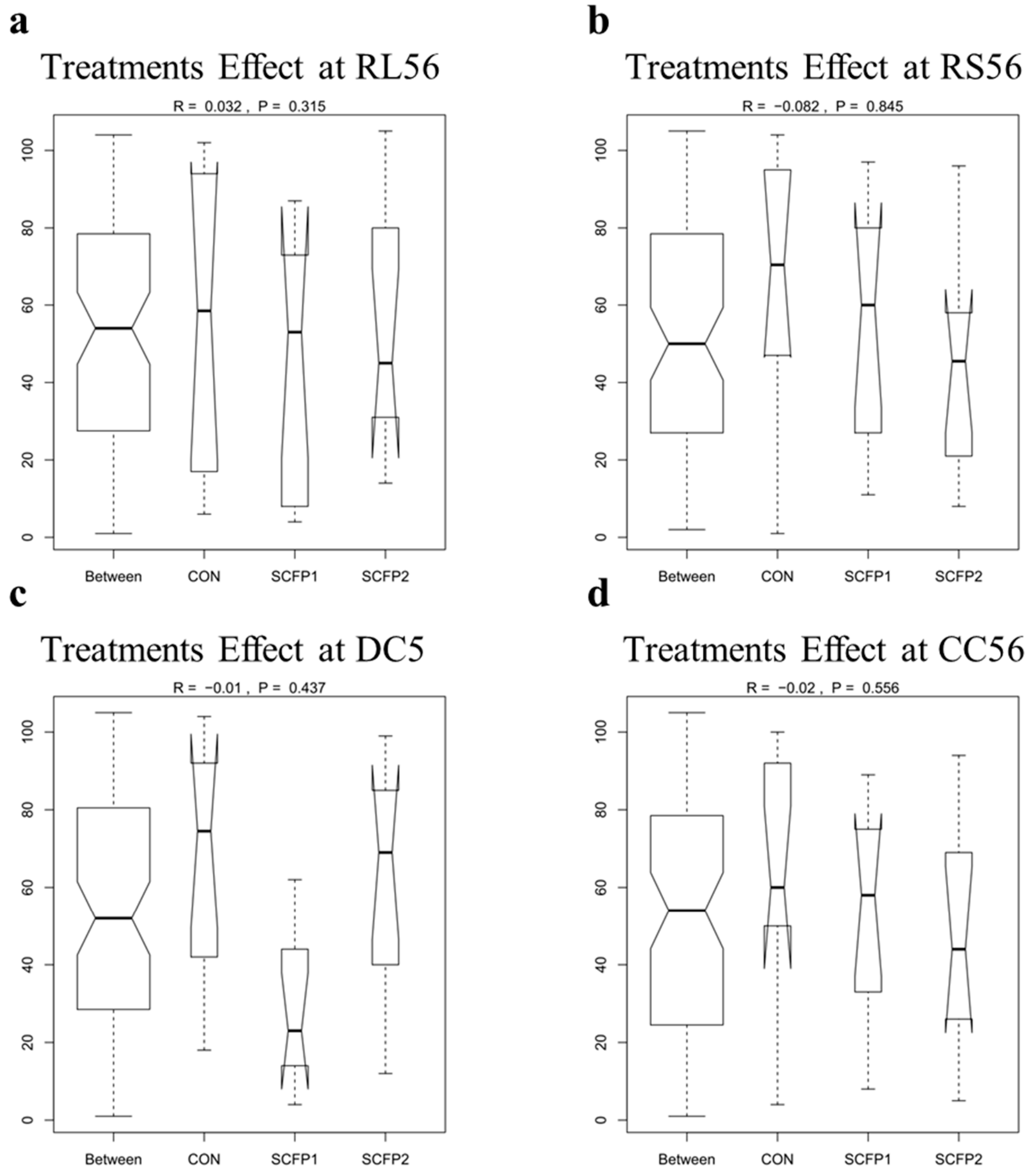

Figure S2. ANOSIM analysis were performed among **t**reatments in RL56 (a), RS56 (b), DC56 (c) and CC56 (d).

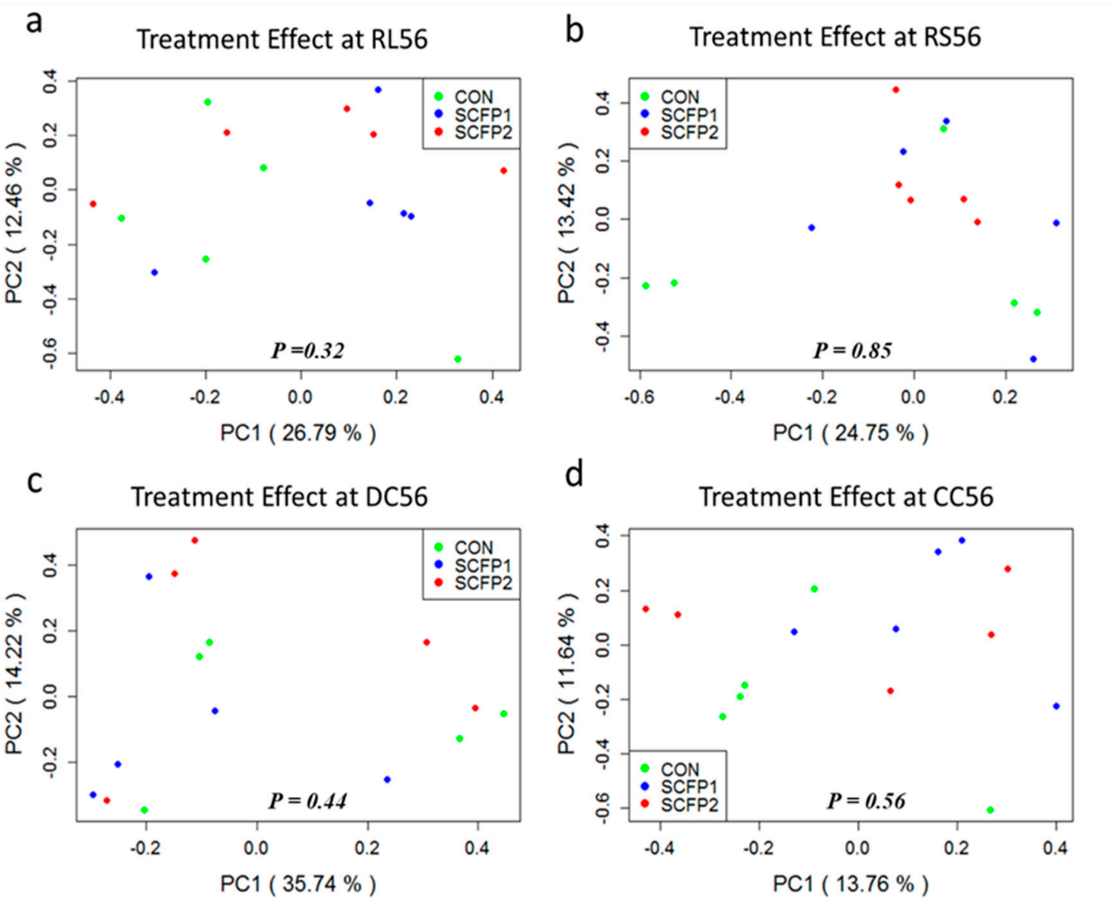

Figure S3. Separation of samples based on phylogenetic information using PCoA plot. (a), treatment separation in RL56. (b), treatment separation in RS56. (c), treatment separation in DC56. (d), treatment separation in CC56.
